# Year-Book of American Contributions to Medical Science and Literature

**Published:** 1860-11

**Authors:** 


YEAR BOOK OF AMERICAN CONTRIBUTIONS TO MEDICAL SCIENCE AND LITERATURE.—Dr. O. C. Gibbs, of Frewsburg, Chautauque County, New York, has issued a circular, stating that if he receives the necessary encouragement he will prepare a yearly volume with the above title, containing from five hundred to eight hundred pages, the first volume to appear early in 1861. The design of the work is to furnish a complete and classified summary of, and index to, all the important contributions found in the medical journals of this country, as well as in the published proceedings of the National and the various State Medical Societies for the preceding year. It will also contain reviews of all medical works of American authorship, abstracting all novelties in practice or opinion contained in them; and a copious index will complete the work. As Dr. Gibbs does not intend to publish the volume at a pecuniary loss, he requests that subscribers will send in their names at once, the payment of three dollars to be made on its appearance. He also requests that publishers of journals and medical works, authors, and presidents of societies, will be kind enough to send him copies of their publications, which he will reciprocate by an exchange.

